# Supplementary material for: Patients want to be seen: The top 3 information needs of patients with inguinal hernia
Source: PLoS One. 2020 Oct 13;15(10):e0240433. doi: 10.1371/journal.pone.0240433 (PMC7553313; doi:10.1371/journal.pone.0240433)
Supplement: Attachment — Submitted filename: reply_plos_one_CO.docx [file pone.0240433.s001.docx]

Dear editor,

Thank you for the opportunity to revise and improve our manuscript

We are grateful for the good quality review comment. These comments definitely improves the article. Unfortunately, because of the hectic period with the COVID-19 we were unable to send in a reply before.

As we revised both your and the reviewer’s suggestions, a point by point description of the revised parts are given per reviewer.

**Editor**

I am sorry for the delay in processing this submission. I was not the original assigned editor. Please do give careful consideration to the reviewer's comments. I also have a query about language. I presume the interviews were originally done in Dutch, and have been translated into English. You should describe the translation process and how you tried to assure accuracy. I note that on two occasions, your interviewees are said to have referred to spinal anaesthesia calling it a "spinal tap." The correct term for spinal anaesthesia is a spinal block. (There are also more complicated terms for it.) "Spinal tap" refers to the extraction of cerebrospinal fluid for diagnostic purposes, a completely different procedure. If the interviews were done in English and the respondents did in fact say that, you should point out that they are in error. If these were translated, this is a mistranslation which leaves me uncertain as to the translation quality in general. The English in the text is generally readable, but at times stilted and there are some odd word choices. Please have this reviewed by a native or fully fluent English speaker. Also, the title refers to 2020 but this research was done in 2019 or earlier. You should delete the year from the title.

*Thank you for your comments. We adapted the manuscript accordingly, changed the title, and had the manuscript checked by a native speaker. We thank both the editor and the reviewer for their comments as we believe that they greatly improved our paper.*

**Reviewer 1**

The authors interviewed patients who had or were scheduled for a hernia surgery on the use of a 360 video of their surgery. Given the scope of patient interest in being engaged in their treatment as well as growing support for technology to support patients’ needs, this study is both timely and unique. The study was simple in design and thoughtfully conducted. The findings reflect themes elaborating the value of the innovative nature of the intervention the utility of supporting patients in non-traditional ways to improve follow-up and experience.

*We thank the reviewer for these encouraging comments, and the comments as we believe that they greatly improved our manuscript.*

Abstract:
Please include some brief patient demographics

*We added some patient demographics, also to the manuscript itself*

Introduction:
The background is simply insufficient. What is the context for this work. What else has been done. Why is this work important in the field, and for patients and physicians?
More discussion around patients involvement in treatment would be useful. Also, this is such an important area of innovation and thinking, the authors should talk at some depth around the advancement of technology in medical decision making and patient-centered care.

*We agree with the reviewer that the introduction was a bit (too) short and therefore rewrote the introduction.*

Method:
Some further elaboration on what “coding” is, and how the process of coding was completed. In other words, how did the authors get from “coded” elements in the transcripts to “themes”. How were codes prioritized, how was meaning ascribed to the codes, and in what ways did inductive vs deductive approach assist in the process (bottom up vs top down). Essentially, a bit more explanation here in the way of definitions, an example, or a description of an inductive vs deductive code would be useful for non-qualitative researchers. In that way, a definition or explanation of a “coding tree” versus a concept map, might be helpful.

*We have added elaboration on the coding process by describing more specifically how the coding process was organized and how inductive and deductive coding was used. In addition, more explanation was given about the coding tree.*

Results:
There seem to be three main themes or groups of themes presented. The authors could organize their results a little more to give more life to these important findings. I will try to illustrate this need below.
seeing”
The set of findings (themes 1 and 2) would benefit from a bit more description in the themes or in the description of themes. What I mean to say is that the themes all link to the “visual tool” and would be more descriptive if they could reflect that connection, i.e., “Visual tool helped patients be seen as unique and partner in treatment process.”

A little more organization within the themes (1 and 2) would help pull the findings together around the two main ideas - being seen (literally) and being seen (figuratively). While these are generally okay, they obfuscate the subtle and arguably more important differences between them. In my view, your argument about being seen (visually) reflects a more personal (authentic) connection with the provider. The other theme, being seen (figuratively) seems to reflect more of a value of being trusted or being viewed as a partner or equal in the treatment process. While I recognize that the authors’ use of literal and figurative are correct, I worry in some way that their close association makes it harder to see the valuable differences in the themes.
Then there is the third theme. The third theme, or category of themes, “Seeing is understanding” is very revealing and offers important insight into the patient experience. I worry that because the authors do not organize the results in a way to prepare the reader for three larger themes with sub-themes, this final section loses its punch.

*To put more emphasis on the third theme we introduced the three themes more clearly, and restructured all themes by renaming themes, removing quotes and rephrasing and shuffling paragraphs.*

The authors could elaborate a bit more using their own narrative between illustrative quotes. While very helpful, the quotes lose their strength when grouped together. In other words, subtlety in the quotes should be drawn out with the use of descriptive narrative. Or if the quotes seem to reflect the same idea, it would be better to pick one quote rather than use two or three to convey the same idea.

*We carefully discussed all the quotes and checked which one were duplicates and removed them.*

Given the work conducted by the authors to “identify, label, and code” the transcripts, it is hard to believe that the themes “emerged” from the transcripts. I believe the authors did lots of work to examine the text and make well-intentioned choices when they derived the final “themes”. The section would be improved if authors could use a verb other than “emerged” to describe how the “themes” were generated by a thorough process of coding, analysis, and achieving consensus.

*The reviewer is correct. During the coding and analyses phase it became clear that many patients literally used the word seeing (or being seen etc) many times and that many of the other factors were relating to this same ‘overall topic”. We therefore reprased the introduction of the result section.*

We hope that this revision will be followed by further reviewing/ publication of our article and are looking forward to hearing your response.

Yours sincerely, on behalf of all co-authors,

Karlijn van Stralen PhD
