# Supplementary material for: Patients want to be seen: The top 3 information needs of patients with inguinal hernia
Source: PLoS One. 2020 Oct 13;15(10):e0240433. doi: 10.1371/journal.pone.0240433 (PMC7553313; doi:10.1371/journal.pone.0240433)
Supplement: Attachment — Submitted filename: 2reply_plos_one_20200923_def.docx [file pone.0240433.s002.docx]

We thank the reviewer for his/her comments. We believe that both the previous and the current comments improved the papers and hope that the paper is now suitable for publication.

Kind regards,

On behalf of all authors

Karlijn van Stralen

Abstract
- I feel that the abstract is missing the actual conclusions. The results are stated but the implications of the findings are not clear.
*We changed the abstract and make a division between the results and the conclusion.*

Conclusion: In order to address to patients’ information needs, complementary tools or services are needed that increase personal contact as well as tailoring with respect to individual needs. Even though video-apps are a partial alternative, hospitals should still offer patients the possibility for face-to-face meetings with physicians as this is highly valued by patients and for instance leads to increased trust in physicians’ performance.

Introduction
- Suggest review for minor typos (extra spaces, missing commas, “postpone” does not need to be hyphenated)

*We reviewed the paper and adapted it accordingly*

- The items in this sentence don't seem to be mutually exclusive: "However, it is not always clear what information patients want, as some prefer wide-ranging information (4, 5), whilst others prefer detailed information (4, 6)." Perhaps by wide-ranging, you mean more general information?

*We changed it to the following:*

*“*However, it is not always clear what information patients want as some prefer receiving very general information about just the procedure (4, 5), whilst others prefer detailed information for example on outcomes and survival (4, 6).”

- For this sentence, “Currently, patients have access to the Internet, which offers unlimited amounts of health information(7).” Suggest changing ‘currently’ to ‘most’ – not all patients have internet access.

*Changed accordingly*

- I’m not sure how I feel about this sentence: “In our hospital we had the impression that patients were missing certain information and that they did not read or (fully) understand the provided (written) information (personal opinion).”

*We removed the word “personal opinion”. We want to indicate that we noted that patients had not read or understood the provided information as they for example had forgotten to take medication prior to the surgery, even though this was written in the instructions. Therefore we wanted to see whether we could improve the understanding of the procedure and the process.*

- The entire paper is about the provision of a virtual reality tool but there is only one sentence about VR in the introduction. This seems off – I would suggest adding more on the evidence and introduction of this concept.

*We added more information on the use of VR to the introduction.*

*“*Virtual reality has been used to train physicians or residents to improve their communication skills towards the patient (11). Furthermore, one study suggested that informing patients on a surgical procedure via VR might improve patient comprehension of their condition (12).”

Methods
- The authors state the used COREQ however they did not include a copy of the completed checklist as a part of their submission. Perhaps not a requirement for the journal but it strengthens the submission.
- Most methods section is written in the passive voice. This is a choice but the manuscript would be much more acceptable to a lay reader if it was written in the active voice.

*We made the method section more “active”.*

- Most of the information provided under the subheading ‘Participants’ is really material that should be reserved for the results section. This includes all of the information starting with the sentence “In total, 21 patients were contacted…” through the end of this subsection. Along with the information in the next section on the length of the interviews.

*We changed this accordingly*

- I think there is a word missing from this sentence: “This 360 ̊ video offered the patients to virtually experience some parts of the operation day and to see some real operation scenes of a laparoscopic inguinal hernia repair procedure.”

*We changed the sentence*

“In this 360˚ video patients could virtually experience parts of the day of the surgery and see parts of a laparoscopic inguinal hernia repair procedure.”

- Given that the themes in the results include in-person requests, I would suggest adding the dates of these interviews due to the COVID-19 shifts that you acknowledge in the conclusions.

*We added the period to the methods section (study design)*

Interviews were held between September and December 2018.

- I see no mention of data saturation which is one of the COREQ checklist items and is important in qualitative research. The authors mention that they started coding before interviewing was complete so perhaps this was in fact done?

*We added the COREQ checklist and added information on when the data collection was ended (methods – data collection)*

The data collection was ended after saturation was reached.

Results
- I’m not following the connection between the subheading ‘visual tools’ and the content under it – I

don’t see references to visual tools here?

*We changed the subchapter title to Using tools*

- The authors seem to have done a nice job responding to previous reviewers comments about breaking up the quotes and improving the clarity of the themes.

*We thank the reviewer for his/her nice comments – we believe that the comments of the reviewer strongly improved our paper*

- I would highly suggest a table to layout the major themes from the analysis. With only one table in the manuscript at present, this could really strengthen the summarization of the findings.

*We added a table 2.*

- I’m don’t think the word ‘sparring’ is used correctly in this sentence: “For others, the Internet was not perceived as helpful in providing sufficient confidence for an active role as a sparring partner.”

*We changed the wording to “for an active role in the share d decision making process”*

- You mention in the abstract that the patients did not find the VR fearsome but I don’t see this as a theme in the results. Perhaps I missed it? I was sort of looking for a heading on the acceptability of the VR but perhaps not enough information emerged on this?

*The VR video was strongly accepted, and not of the patients found it troublesome. There was too little information to have an entire theme on this topic. However, we combined the finding from various places to the end of the results section*

Still, the use of the VR-video was accepted by all patients and none of the patients found it fearsome themselves. Beforehand we had expected that patients might find it fearsome to view a surgical procedure in 360. Some of the patients indicated that it could scare people off to get so close to the real surgery, although they did not find it problematic themselves. In addition there was one patient who indicated not to be interested in the video and who had the interview by phone.  

Discussion
- You say “these studies” but then only one citation, which I see is a systematic review. Perhaps you could say instead introduce citation 14 as a 2018 systematic review and then in the next sentence say “the studies in this review were mainly conducted…”

*We changed the wording*

However, most of the studies included in this review were conducted before 2010.

- This sentence sits at the end of a paragraph without further explanation: “Alternative options could be for example use of autobots.”

*We added some literature on this.*

Alternative options could be, for example, use of chatbots. For the latter, studies in breast cancer patients, for example, have shown that an artificial conversation agent achieved equal satisfaction scores as “real” physicians (18).

I feel this contextual paragraph needs improvement. Surely there are other results in the literature that indicate if your findings are in agreement with other studies that patients felt there was a lack of tailored information?

We agree and added some more information and results from the literature

Patients felt that the general information obtained from the brochures, the internet or doctors assistants, was not sufficiently tailored to their personal situation, for instance for questions regarding their recovery process. It had been shown previously that tailored information provided on the internet leads to higher satisfaction rates among patients, especially among younger patients, although no improvements in patients outcomes have been found (17,18). Some of our patients had WhatsApp contact with their surgeon, which was highly appreciated. However, this would be nearly impossible to provide to all patients, as physicians are often afraid of being overloaded with questions from patients. Still research has shown benefits of a short messaging system protocol leading to a reduced number of clinic visits and increased efficiency of clinic visits(19). Alternative options could be for example use of chatbots. For the latter, studies in breast cancer patients, for example, have shown that an artificial conversation agent achieved equal satisfaction scores as “real” physicians (20).

- When I read sentences like: “The majority of the patients believed that the 360 ̊ video enhanced their understanding, especially with regard to the surgical procedural information.” In the discussion, I feel like this is missing from the results. Perhaps I missed it but again I do wonder if a section about the acceptability of the 360 tool would be appropriate in the results section.

*See above.*

- In the section under “seeing is understanding” – is there other research around VR that could be incorporated so that you are reviewing your results against the current literature on VR?

We added some literature

Also in other fields it has been suggested that VR can help in pharmacy education by providing immersive educational activities (24), having elderly patients coping with dementia (25) or teaching skills to health professionals (26).

- I think you can remove “for instance” from the last line of the manuscript.

- *Adapted accordingly*
